# Cardiac Biomarkers and Left Ventricular Hypertrophy in Asymptomatic Hemodialysis Patients

**DOI:** 10.3889/oamjms.2016.011

**Published:** 2015-12-30

**Authors:** Reneta Yovcheva Koycheva, Vasil Cholakov, Jivko Andreev, Margarit Penev, Rosen Iliev, Krasimira Nancheva, Vanya Tsoneva

**Affiliations:** *Department of Nephrology, University Hospital “Prof. Dr. Stoyan Kirkovich”, Stara Zagora, Bulgaria*

**Keywords:** hemodialysis, cardiac troponin T, natriuretic peptide, C-reactive protein, left ventricular hypertrophy

## Abstract

**BACKGROUND:**

Cardiac biomarkers are often elevated in dialysis patients showing the presence of left ventricular dysfunction. The aim of the study is to establish the plasma levels of high-sensitivity cardiac troponin T (hs TnT), precursor of B-natriuretic peptide (NT-proBNP) and high sensitivity C-reactive protein (hs CRP) and their relation to the presence of left ventricular hypertrophy (LVH) in patients undergoing hemodialysis without signs of acute coronary syndrome or heart failure.

**MATERIAL AND METHODS:**

We studied 48 patients - 26 men and 22 women. Pre and postdialysis levels of hs cTnT, NT-proBNP and hs CRP were measured at week interim procedure. Patients were divided in two groups according to the presence of echocardiographic evidence of LVH - gr A - 40 patients (with LVH), and gr B - 8 patients (without LVH).

**RESULTS:**

In the whole group of patients was found elevated predialysis levels of all three biomarkers with significant increase (p < 0.05) after dialysis with low-flux dialyzers. Predialysis values of NT-proBNP show moderate positive correlation with hs cTnT (r = 0.47) and weaker with hs CRP (r = 0.163). Such dependence is observed in postdialysis values of these biomarkers. There is a strong positive correlation between the pre and postdialysis levels: for hs cTnT (r = 0.966), for NT-proBNP (r = 0.918) and for hs CRP (r = 0.859). It was found a significant difference in the mean values of hs cTnT in gr. A and gr. B (0.07 ± 0.01 versus 0.03 ± 0.01 ng/mL, p < 0.05) and NT-proBNP (15,605.8 ± 2,072.5 versus 2,745.5 ± 533.55 pg/mL, p < 0.05). Not find a significant difference in hs CRP in both groups.

**CONCLUSIONS:**

The results indicate the relationship of the studied cardiac biomarkers with LVH in asymptomatic patients undergoing hemodialysis treatment.

## Introduction

Cardiorenal syndrome type 4 often manifests itself with patients undergoing hemodialysis treatment in stage 5 HD on chronic kidney disease (CKD). It is characterized by primary CKD, associated with left ventricular (LV) damage, including left ventricular hypertrophy (LVH), systolic and diastolic dysfunction and congestive heart failure [1]. Echocardiographically-proven LV hypertrophy is detected in 60-75% of patients starting renal replacement therapy and in 60-90% of those on regular dialysis treatment [2, 3] LVH is considered an independent risk factor for cardiovascular mortality in this population [4-6]. Pathophysiologic factors involved in LVH of CKD and ESRD patients have generally been divided into 3 categories [7-9]: (1) related to afterload, (2) related to preload, and (3) not related to afterload or preload. The ones in the first category are represented by an increase in systemic arterial resistance, elevated arterial blood pressure, and reduced large-vessel compliance [7-9] related in part to aortic ‘calcification’, which is typical in CKD patients; all these factors result in myocardial cell thickening and concentric LV remodeling often together with activation of the intracardiac renin-angiotensin system [9, 10]. Additional roles have inadequate activation of the renin-angiotensin-aldosterone system, hypervolemia, anemia, oxidative stress and inflammation [4, 11, 12]. Oxidative stress and xanthine oxidase activation as well as the phosphodiesterase-5 pathway may also be involved in the development of LVH [13]. Among the preload-related factors, the role of intravascular volume expansion (salt and fluid loading) has to be underlined, as well as secondary anemia and the presence of arterovenous fistulas [14, 15], resulting in myocardial cell lengthening and eccentric or asymmetric LV remodeling. Both afterload- and preload-related factors operate with additive and synergistic effects. As a result, myocardial hypertrophy induces the activation of cellular apoptotic signals and activates metabolic pathways able to increase extracellular matrix production up to fibrosis [16, 17]. Fibrosis leads to progressive impairment in contractility with stiffening of the myocardial wall, systolic and diastolic dysfunction, dilated cardiomyopathy and congestive heart failure [18]. Renin-angiotensin-aldosterone system activation induces hyperaldosteronemia promoting cardiac fibrosis through the generation of signals leading to profibrotic transforming growth factor production [19]. The LVH can also be promoted by iron and/or erythropoietin [20] or vitamin D deficiency [21]. The presence of arterovenous fistulas can contribute to the development of LVH because of excess blood flow that increases the myocardial workload [22]. Persistent inflammatory condition is recognized as an important risk factor for the development of cardiovascular complications in this group of patients. Their levels of C- reactive protein (CRP) correlate positively with LVH [23]. Cardiac biomarkers reflect LV structure and function and are essential for early diagnosis of heart failure. In recent years, natriuretic peptides, in particular the B-type natriuretic peptide (BNP), are used in screening for the risk of cardiovascular events in the general population [24-27]. BNP is synthesized in ventricular myocytes, undergoing intracellular metabolism to prohormone (proBNP), which is then fragmented into activated (C-terminal) and inactive (N-terminal) proBNP. In practice, NT-proBNP is tested more often because of its longer half-life in the circulation (60-120 min) than that of BNP, which is 23 minutes long [24]. BNP and NT proBNP are biological markers of left ventricular dysfunction [28]. Another biomarker is cardiac troponin T (cTnT), which is released into the circulation and increases sharply if irreversible damage to the heart muscle exists [29]. There are other reasons for the presence of cTnT. These are myocardial damage due to high pressure in the left ventricular wall in hypertrophy, acute or chronic volume overload, microvascular lesions, “quiet” subclinical myocardial ischemia fibrosis and necrosis. In hemodialysis patients, these conditions are very common [30].

Objectives of the study are: -to determine plasma levels of high-sensitivity cardiac troponin T (hs sTnT), NT-proBNP and high sensitivity C-reactive protein (hs CRP) and their changes during hemodialysis; and -to link LVH and cardiac biomarkers in patients undergoing hemodialysis without signs of acute coronary syndrome or heart failure.

## Material and Methods

Forty eight patients were studied (26 men and 22 women) undergoing a standard 4-hour bicarbonate dialysis three times a week, over three months. All patients used low-flux dialyzers with polysulfone membranes. The adequacy of dialysis was evaluated by URR%. Clinically they are hemodynamically stable (in sinus rhythm, with values of BP below 150/90, without manifestation of hypertensive crisis, without clinical manifestations of hypotensive reactions), with no evidence of acute coronary syndrome or heart failure in the previous two months. Patients with a diagnosis of cancer, autoimmune diseases and signs of active inflammation were excluded.

Determined pre- and post-dialysis are levels of hs sTnT, NT-proBNP and hs CRP immediately before the interim procedure for the week. hs sTnT and NT-proBNP were analyzed with Elecsys 2010 immunoassay reagents of Roshe Diagnostics. NT-proBNP was reported in pg/mL. The values of NT-proBNP > 125 pg/mL are considered a threshold taken from the manufacturer’s package insert above which it can be assumed cardiac dysfunction related to an increased risk of cardiac events (myocardial infarction, heart failure, death). The above hs sTnT reference value is specified by the manufacturer through testing of 533 healthy subjects (99th percentile of healthy) and 0.014 ng/mL. hsCRP was investigated by biochemical analyzer Beckman Coulter AU 480 by immunoturbidimetric method with reagents Roshe Diagnostics and has an upper reference value of 1.0 mg/L. Patients were divided into two groups according to the presence of the echocardiography evidence of LVH - group A (with LVH) and group B (without LVH). Echocardiography was performed on non-dialysis day, 24 hours after the last hemodialysis, with PHILIPS XE 11.

Left ventricular structure was evaluated by 2M echograph mode. LV mass was corrected for body surface area and presented as LV mass index (LVMI). The criteria for LVH are LVMI > 115 g/m^2^ in men and LVMI > 95 g/m^2^ in women. Compared were the thickness of the free wall of the LV (LVPW d), the thickness of the interventricular septum (IVS d) in diastole, the ejection fraction (EF%) and fraction of shortening (FS%) in both groups of patients. All patients were in sinus rhythm with no abnormalities in the kinetics of the LV wall and without severe valvular heart damage.

To process the statistical data was used R-Project software version 3.1.1. Descriptive statistics was presented and statistical results are reported in mean ± standard error of the average. All parameters were tested for normal distribution using the test of Shapiro-Wilk. The relationship between performances was recorded using linear regression. Statistically significant difference was accepted at p < 0.05.

## Results

The main demographic and clinical characteristics of the study group of 48 patients were: 26 men and 22 women; mean age 53.04 ± 2.24 years; the average duration of hemodialysis treatment 54 ± 7.74 months; the average URR% - 64.8%. In 17 patients the primary renal disease leading to CKD is chronic tubulointerstitial nephritis, in 14 - chronic glomerulonephritis, in 9 - diabetic nephropathy, in 5 - polycystic kidney disease and three - other disabilities (congenital anomalies, obstructive nephropathy).

In all of the patients were found elevated mean values of cardiac biomarkers and hs CRP, both before and after the dialysis procedure. Post-dialysis levels of hs cTnT and NT-proBNP were significantly higher (p < 0.001) while hs CRP significantly lower (p < 0.05). A statistically significant difference was registered as well as a very high correlation between pre- and post-dialysis levels of markers tested: in hs cTnT (r = 0.966), in NT-proBNP (r = 0.918) and hs CRP (r = 0.859) – the statistical analysis in both groups of patients is based on predialytic values. (Table 1) Predialytic indicators studied in men and women showed significant difference in hs cTnT (0.07 ± 0.02 compared to 0.06 ± 0.01; p <0.05). The remaining two biomarkers are higher in women, but the difference was not statistically significant (Table 2).

**Table 1:** Values of cardiac biomarkers before and after the dialysis procedure

Biomarker	Predialytic values of biomarkers mean ± sem	Postdialytic values of biomarkers mean ± sem	P	r
hs cTnT (ng/mL)	0.06 ± 0.01	0.07 ± 0.01	p < 0.001	0.966
NT-proBNP (pg/mL)	13,462 ± 1,862	15,279 ± 1,895	p < 0.001	0.918
hs CRP (mg/L)	16.30 ± 4.19	15.44 ± 3.73	p < 0.05	0.859

**Table 2:** Predialytic values of cardiac biomarkers in men and women

Value	Men	Women	P
hs TnT	0.07 ± 0.02	0.06 ± 0.01	p < 0.05
NT-proBNP	12,845 ± 2,624	14,192 ± 2,683	p - NS
hs CRP	15.21 ± 6.35	17.65 ± 5.29	p - NS

Predialytic values of NT-proBNP in all investigated patients showed moderate correlation with hs cTnT (r = 0.47) and a weaker one with hs CRP (r = 0.163). Such dependence is observed in the post-dialysis values of these parameters, respectively compared hs cTnT (r = 0.482) and hs CRP (r = 0.19) (Table 3).

**Table 3:** Correlations between the values of NT-proBNP with hs cTnT and hs CRP - before and after hemodialysis

Predialytic values of biomarkers	Correlation coefficient (r)	Postdialytic values of biomarkers	Correlation coefficient (r)
hs cTn T	0.47	hs cTn T	0.482
hs CRP	0.163	hs CRP	0.19

Forty patients from group A (with LVH) take part in the echocardiographic study, while in group B (without LVH) - 8 patients participate: LVH was present in 83% of the study group. Table 4 shows the ultrasound data of the two groups. Patients with LVH had significantly higher values of LVMI, LVPW and IVS, while indicators of systolic function EF% and FS% showed no differences in both groups.

**Table 4:** Echocardiographic parameters in patients with and without LVH

Echocardiographic parameter	Value mean±sem		P
	Group A with LVH	Group B without LVH	
LVMI g/m^2^	151.97 ± 4.23	94.24 ± 5.27	p<0.005
LVPW d/sm	1.19 ± 0.02	0.94 ± 0.06	p<0.005
IVS d/sm	1.34 ± 0.03	1.08 ± 0.05	p<0.005
EF%	62.8 ± 1.58	68.5 ± 3.29	p - NS
FS%	35.2 ± 1.17	38.4 ± 2.4	p - NS

With all patients studied, levels of NT-proBNP were significantly higher than the threshold values. In group A the average is 15,605.8 ± 2,072.5 pg/mL with 77.5% having values above the limit of chronic heart failure functional class I NYHA (3,410 pg/mL). In group B the recorded average value of this biomarker is 2,745.5 ± 533.6 pg/mL, with only 25% of the patients with the values were above the threshold for chronic heart failure functional class I NYHA. A significant difference was found in the mean values of hs cTnT between patients in group A and B (0.07 ± 0.01 ng/mL compared to 0.03 ± 0.01 ng/mL, p <0.05) and NT- proBNP (15,605.8 ± 2,072.5 compared to 2,745.5 ± 533.6 pg/mL, p < 0.05) before hemodialysis. Although the values of hs CRP were higher in patients with LVH, there wasn’t a significant difference in this indicator in both groups. A similar dependence was observed in post-dialysis values of biomarkers (Table 5).

**Table 5:** Pre- and post-dialysis values of cardiac biomarkers in the two groups of patients

	Group A with LVH	Group A without LVH	P
**Predialytic values of biomarkers**			
hs cTnT	0.07 ± 0.01	0.03 ± 0.01	p < 0.05
NT-proBNP	15,605.8 ± 2,072.5	2,745.5 ± 533.6	p < 0.005
hs CRP	17.87 ± 4.85	7.35 ± 4.05	p=0.054
**Postdialytic values of biomarkers**			
hs cTnT	0.08 ± 0.01	0.04 ± 0.01	p < 0.05
NT-proBNP	16,807.7 ± 2,099	7,635 ± 3,455.9	p < 0.05
hs CRP	16.66 ± 4.39	9.5 ± 4.59	p - NS

Statistical analysis established that there was a positive moderate correlation between hs cTnT and NT proBNP (r = 0.43). of hs cTnT with hsCRP (r = 0.61). and a weaker. but also positive correlation between NT proBNP and hsCRP (r = 0.10) in patients with LVH. In this group there is a positive correlation of each of the biomarkers with LVMI. LVPW IVS and it is most pronounced compared to the thickness of the interventricular septum (Table 6). Three cardiac biomarkers showed negative correlation with EF% and FS%.

**Table 6:** Correlation analysis between cardiac biomarkers and echocardiographic parameters in patients with LVH

	hs cTnT	NT proBNP	hs CRP	LVMI	LVPW	IVS	EF%	FS%
**hs cTnT**	-	r=0.43	r=0.61	r=0.23	r=0.28	r=0.46	r= -0.04	r=0.001
**NT-proBNP**	r=0.43	-	r=0.10	r=0.31	r=0.24	r=0.32	r=-0.21	r=-0.21
**hs CRP**	r=0.61	r=0.10	-	r=0.18	r=0.39	r=0.50	r= -0.25	r= -0.08

## Discussion

Our study found a very high prevalence of LVH in hemodialysis patients, i.e. 83%. Similar results presented by other studies indicate that 75-78% of these patients had LVH in stage 5 CKD on HD [31, 32]. We found a positive correlation of hs CRP with ultrasound parameters LVMI. LVPW and IVS (r = 0.18; r = 0.39; r = 0.50) in group of patients with LVH. This, on its own part, defines the essential role of inflammation in the development of LVH in patients on hemodialysis. Along with hypertension and volume overload, systemic inflammatory state may contribute to the development of LVH by changing the structure and function of vascular smooth muscle cells. leading to increased vascular stiffness [33]. On the other hand, it is considered that chronic subclinical inflammation alters LV structure and geometry by disrupting the balance between cell growth and apoptosis in cardiac tissue [34].

When using a low-flux dialyzers. there was a significant increase in the levels of NT-proBNP in all investigated patients. Being released into the circulation in abnormally high volume, it stretches cardiomiofibres. The established increased level after an adequate hemodialysis procedure and normal hemodynamics, suggests the role of other factors in increased plasma levels. Wahl et al [35] reported that pre- and post-dialysis levels of NT-proBNP are dependent on the type of membrane used during HD. High-flux membranes have a higher ultrafiltration rate than low-flux membranes. They tend to have larger pores, which means that the clearance of NT-proBNP with a molecular weight of 8.5 kDa is higher than when using low-flux membranes. where the post-dialysis concentration increased. In our study we found that NT proBNP was, to a high degree, correlated with some echocardiographic parameters – LVMI, LVPW and IVS in group of patients with LVH. This biomarker of cardiomyocyte microinjury and hemodynamic stress may stimulate fibrosis-related mechanisms and facilitate the diagnosis of subclinical LV remodelling and LVH in this population.

hs cTnT values above the 99th percentile in healthy frequently found in patients with LVH and heart failure in the absence of acute coronary syndrome and in patients with CKD and without clinical heart disease [36-38]. The mechanism of this subclinical myocardial injury with elevated sTnT has not yet been elucidated [39]. It is believed that stretching and deformation of myocardium in LVH impairs the permeability of cardiomyocytes, leading to the release of cardiac troponins. On the other hand, the increased oxygen demand in hypertrophied LV with reduced coronary reserve may cause subclinical ischemia with or without macrovascular coronary artery disease [39]. Since all patients studied are asymptomatic, the established moderately high positive correlation between LVMI, LVRW and IVS and the values of the cardiac biomarkers suggest that their measurement can be used for preclinical diagnosis of LVH.

In conclusion, our data is based on 83% incidence of LVH in patients receiving dialysis. Pre- and post-dialysis values of cardiac biomarkers and hs CRP were increased in all patients just as it is manifested in those with LVH. There is a positive. moderate correlation between the degree studied biomarkers. Cardiac biomarkers correlate well with echocardiography parameters showing LVH. The study of cardiac biomarkers can be used as a screening for the presence of LVH in asymptomatic patients treated with hemodialysis.
